# Beneficial Effects of Cod Protein on Inflammatory Cell Accumulation in Rat Skeletal Muscle after Injury Are Driven by Its High Levels of Arginine, Glycine, Taurine and Lysine

**DOI:** 10.1371/journal.pone.0077274

**Published:** 2013-10-04

**Authors:** Junio Dort, Nadine Leblanc, Julie Maltais-Giguère, Bjørn Liaset, Claude H. Côté, Hélène Jacques

**Affiliations:** 1 Department of Food Science and Nutrition, Laval University, Quebec City, Quebec, Canada; 2 Institute of Nutrition and Functional Foods, Laval University, Quebec City, Quebec, Canada; 3 National Institute of Nutrition and Seafood Research, Bergen, Norway; 4 Department of Rehabilitation, Laval University, Quebec City, Quebec, Canada; 5 Quebec University Hospital Centre, Laval University, Quebec City, Quebec, Canada; National Institute of Agronomic Research, France

## Abstract

We have shown that feeding cod protein, which is rich in anti-inflammatory arginine, glycine, and taurine, may beneficially modulate the inflammatory response during recovery following skeletal muscle injury; however it is unknown if these amino acids are responsible for this effect. This study was designed to assess whether supplementing casein with an amino acid mixture composed of arginine, glycine, taurine and lysine, matching their respective levels in cod protein, may account for the anti-inflammatory effect of cod protein. Male Wistar rats were fed isoenergetic diets containing either casein, cod protein, or casein supplemented with L-arginine (0.45%), glycine (0.43%), L-taurine (0.17%) and L-lysine (0.44%) (casein+). After 21 days of *ad libitum* feeding, one tibialis anterior muscle was injured with 200 µl bupivacaine while the saline-injected contra-lateral tibialis anterior was served as *sham*. Cod protein and casein+ similarly modulated the inflammation as they decreased COX-2 level at day 2 post-injury (cod protein, p=0.014; casein+, p=0.029) and ED1^+^ macrophage density at days 2 (cod protein, p=0.012; casein+, p<0.0001), 5 (cod protein, p=0.001; casein+, p<0.0001) and 14 (cod protein, p<0.0001; casein+, p<0.0001) post-injury, and increased ED2^+^ macrophage density at days 5 (cod protein, p<0.0001; casein+, p=0.006), 14 (cod protein, p=0.001; casein+, p<0.002) and 28 (cod protein, p<0.009; casein+, p<0.005) post-injury compared with casein. Furthermore, cod protein up-regulated (p=0.037) whereas casein+ tended to up-regulate (p=0.062) myogenin expression at day 5 post-injury compared with casein. In the cod protein-fed group, these changes resulted in greater muscle mass at days 14 (p=0.002), and 28 (p=0.001) post-injury and larger myofiber cross-sectional area at day 28 post-injury compared with casein (p=0.012). No such effects were observed with casein+. These data indicate that anti-inflammatory actions of cod protein, contrary to its effect on muscle mass recovery, are driven by its high levels of arginine, glycine, taurine and lysine.

## Introduction

Skeletal muscle injury leads to changes in tissue morphology and function that may last for several weeks [1,2]. Injury is first characterized by muscle protein catabolism and acute inflammation followed by muscle tissue regeneration [1,3,4]. The progression of muscle repair is likely to be modulated by dietary proteins providing essential and nonessential amino acids, modulating inflammation and protein anabolism. In this respect, feeding cod protein has been found to facilitate skeletal muscle regeneration, notably through improving the inflammatory response [5]. However, the mechanisms explaining the beneficial effect of feeding cod protein on muscle inflammation and repair are not known.

Muscle inflammation after injury results in a rapid early response by neutrophils, followed by sequential increases in ED1^+^ and ED2^+^-macrophages [6]. The latter is pivotal in orchestrating tissue repair and remodelling [1]. However, behind the noteworthy importance of scavenging cell debris for subsequent repair, excessive accumulation of neutrophils and ED1^+^-macrophages may delay the resolution of inflammation [7,8]. In this regard, previous findings in mice reported that immunodepletion of neutrophils and invading macrophages can substantially reduce muscle damages [9,10], indicating that limiting the action of neutrophils and ED1^+^-cells would be beneficial for muscle repair.

Dietary fish protein rich in arginine, glycine, and taurine [11] may have various biological functions in muscle repair, such as an improved resolution of inflammation, owing to its ability to decrease the production of major pro-inflammatory cytokines (TNF-α, IL-6) [12,13], and to limit the accumulation of pro-inflammatory macrophages at the site of injury [5]. In addition, arginine, glycine and taurine have been shown to decrease muscle cell damage in various rodent models of inflammation including endotoxin- and exercise-induced muscle damage by inhibiting the secretion of inflammatory markers, such as TNF-α, IL-1β, IL-6, PGE-2 and by reducing COX-2 expression and reactive oxygen species (ROS) generation [14-20]. Interestingly, arginine and glycine were also shown to regulate inflammatory response by reducing the infiltration of neutrophils and phagocytic macrophages [21-23]. Furthermore, the insulin-sensitizing effect of arginine [24] and taurine [25] might counteract the down-regulation of the insulin-sensitive protein anabolism pathways which characterizes muscle injuries [26], thereby promoting muscle protein synthesis. These beneficial roles of arginine, glycine and taurine, especially in the regulation of inflammation, imply that feeding a diet rich in these amino acids might improve skeletal muscle repair.

This study in rats was primarily designed to assess the effects of a supplementation of casein with an amino acid mixture composed of arginine, glycine, taurine and lysine on the accumulation of inflammatory cell populations following muscle injury. In this respect, rats were fed either casein, cod protein or casein supplemented with arginine, glycine, taurine and lysine to achieve the levels found in cod protein. Arginine, glycine and taurine were chosen because of their well-known anti-inflammatory properties, and lysine was incorporated to avoid any lysine-arginine imbalance. Our first working hypothesis was that adding arginine, glycine, taurine and lysine to casein in order to match their respective levels in cod protein, beneficially modulates the inflammatory response following muscle injury to the same extent as cod protein. Because modulation of inflammation could result in increased regenerative capacity of skeletal muscle, we further hypothesized that adding arginine, glycine, taurine and lysine to casein would impact on both morphological (muscle mass and cross-sectional area) and myogenic markers (MyoD and myogenin) as does cod protein compared to casein in injured tibialis anterior muscle. Through this work in rats, we expect to develop a better understanding of how dietary cod protein and its bioactive components can influence skeletal muscle repair after an injury.

## Materials and Methods

### Ethics Statement

This research protocol was approved by the Laval University Animal Care Committee in accordance with The Canadian Council on Animal Care guidelines (Permit Number: 2010046-1). There were no adverse effects observed in locomotion or health status.

### Animals and experimental design

The muscle injury protocol used in the present study is a well-recognized and relevant animal model to study inflammatory response and skeletal muscle regeneration after an injury [1,27]. Male Wistar rats were obtained from Charles River (St-Constant, Quebec, Canada) and were weighing 50-60 g on arrival. They were housed individually in plastic cages maintained at 20°C (45-55% of humidity) with a 12 h/12 h light–dark cycle. After a 7-day adaptation provided by feeding a ground non-purified diet (NPD) (Rodent Chow, Ralston Purina Inc., Lasalle, Quebec, Canada), 120 rats were randomly assigned to three experimental diets (*n* = 40 rats per dietary group). They were then gradually transferred to their respective experimental purified diet (ED) differing in protein source by feeding a mixture of ground NPD and ED over a 4-day period (100% NPD for day 1, 75% NPD and 25% ED for day 2, 50% NPD and 50% ED for day 3, 25% NPD and 75% ED for day 4). Animals had free access to experimental diets and water during the experimental period (including 21 days pre-injury and 28 days post-injury). Food intake and body weight were recorded every two days.

### Experimental purified diets

Powdered purified diets varying in protein source were formulated to provide 20% protein, 15% fat and 58% carbohydrates. The protein source was either casein (89% protein), or cod protein (93% protein) or casein-plus (casein+, 92% protein). Cod protein was delipidated and ground in our laboratory from frozen cod fillets as previously described [5]. The casein+ diet was obtained by supplementing the casein diet with L-arginine (0.45%), glycine (0.43%), L-taurine (0.17%) and L-lysine (0.44%) in order to reach the same levels of these amino acids as in the cod protein diet. The amino acid composition of each protein source is shown in Table 1. The level of protein in the purified diets (casein, 22.5% casein; cod protein, 21.6% cod protein; casein+, 20.5% casein + 0.45% L-arginine + 0.43% glycine + 0.17% L-taurine + 0.44% L-lysine) was calculated on an isonitrogenous basis at the expense of cornstarch (32.5% for casein, 33% for cod protein, 32.8% for casein+) and sucrose (20% for casein, 20.5% for cod protein, 20.3% for casein+). The diets contained 10% lard, 4% soybean oil, 5% cellulose, 1% cholesterol, 3.5% AIN-76 mineral mix, 1% vitamin mixture, 0.2% butylated hydroxytoluene, and 0.3% choline bitartrate. All ingredients were supplied by MP Biomedicals (Solon, Ohio, USA), except for lard and soybean oil, which were purchased from local supermarket. Butylated hydroxytoluene (BHT) was added to the experimental diets in order to prevent oxidation of either n-6 (PUFA) in lard or n-6 and n-3 (PUFA) in soybean oil, necessary to meet essential fatty acids requirement of rats [28].

**Table 1:** Amino acid composition of dietary protein sources (g/100 g of amino acids).

**Amino acids**	**C**	**CP**	**C^+^**
Alanine	2.94	6.54	2.64
Arginine	3.20	5.82	5.82
Aspartic acid	7.00	11.57	6.30
Glutamic acid	21.90	16.71	19.71
Glycine	1.61	4.04	4.04
Histidine	2.61	1.95	2.35
Isoleucine	4.89	4.87	4.40
Leucine	9.09	8.59	8.19
Lysine	7.88	10.66	10.66
Methionine	2.76	3.30	2.48
Phenylalanine	4.95	4.08	4.45
Proline	10.34	3.09	9.31
Serine	5.46	4.50	4.91
Taurine	ND	0.89	0.89
Threonine	4.05	4.57	3.65
Tyrosine	5.07	3.48	4.56
Valine	6.27	5.26	5.64
EAA^1^	42.49	43.28	41.82

C, casein; CP, cod protein; C^+^, casein supplemented with L-arginine, glycine, L-taurine and L-lysine; ND, not detected.

Values for casein and cod protein were provided by an analysis of the amino acid composition.

performed by the National Institute of Nutrition and Seafood Research (NIFES, Bergen, Norway).

^1^Sum of essential amino acids (histidine, isoleucine, leucine, methionine, lysine, valine, threonine, phenylalanine).

As expected, experimental diets were isoenergetic, isolipidic and isonitrogenous. The energy content assessed by automatic adiabatic calorimeter (Model 1241; Parr Instruments, Moline, Illinois, USA) was similar between diets (casein, 20.3 kJ/g; cod protein, 19.6 kJ/g; casein+, 20.2 kJ/g). No difference was observed between diets for the percentage of protein tested by the Dumas method (Leco FP-528, ISO 34/SC 5, Ontario, Canada) (casein, 20.5%; cod protein, 19.4%; casein+, 20.4%). In addition, the lipid content measured by an extraction method (Ankom^XT10^ Extractor, Ankom Technology, Macedon, New York, USA) was similar between diets (casein, 12.9% (W/W); cod protein, 12.7% (W/W); casein+, 12.9% (W/W)).

### Myotoxin injury protocol, surgical method and muscle collection

Muscle injury and collection were performed under anesthesia with isoflurane (2.5%/L O_2_) as in our preceding study [5]. For baseline values, anterior tibialis (TA) muscles of both legs were carefully removed (8 rats per dietary group) in the morning at day 21 of feeding. TA was weighed and cut at the half proximal region into two equal parts. For quantification of muscle regeneration and immunohistochemical analysis, one part was coated with embedding medium, fresh frozen in liquid nitrogen-cooled isopentane and stored at -80°C (DLT-21V-85ABA Harris Manufacturing in North Billerica, Massachusetts, USA). The other part was lysed with lysis buffer (1/10) containing 1% of protease inhibitor cocktail which protects the transcriptional factors from undergoing proteolysis. The lysate was centrifuged at 10000 g (10 min, 4°C) and the protein suspension was retained, aliquoted and stored at -80°C until Western blot processing. Rats were euthanized by cardiac exsanguination under anesthesia with inhaled isoflurane (2.5%/L O_2_) (PPC, Richmond Hill, Ontario, Canada) using an Ohmeda Isotec 3 Vaporiser (BOC Health Care, England).

The remaining animals (32 rats per dietary group) were injected with buprenorphine (0.1mg/kg sc) for pain management and anesthetized with inhaled isoflurane (2.5%/L O_2_), avoiding animal stress. A total of 200 µl of bupivacaine (0.5%) (Marcaine, Abbott, Mississauga, Ontario, Canada) was injected at three sites, proximal, half proximal and distal regions, within the TA, to provoke muscle injury. Bupivacaine—a local anaesthetic drug—is used commonly to cause muscle injury and to study muscle regeneration since it has no effect on satellite cells [29]. Muscle damage induced by myotoxic molecules such as bupivacaine involves an inflammatory response, which is dominated by an accumulation of inflammatory cells in the first few hours after their intramuscular injection. With the amount of bupivacaine injected in this protocol, complete regeneration is generally observed within 4-5 weeks post-injection. The contra-lateral muscle was injected with 200 µl of saline and served as *sham*. Rats were returned to their cages after regaining consciousness and were examined daily for general health conditions. Rats were given additional buprenorphine (0.1 mg/kg sc) twice daily until the third day post-injury depending on the day of sacrifice. On days 2, 5, 14 and 28 post-injury, both injured and *sham* TA were removed (8 animals per dietary group/time point) and processed as described above. Time points 2, 5 and 14 were first selected to highlight the effect of dietary proteins on inflammatory cell (neutrophils and macrophages) accumulation at injured sites. According to Pizza et al [30], neutrophils infiltrate rapidly the damaged muscle after the injury, and can remain at high concentrations up to 5 days whereas the number of ED1^+^ macrophages peaks at 5-7 days after the injury and may remain high for several days. In parallel to the increase of ED1^+^ macrophages, occurs the accumulation of anti-inflammatory ED2^+^ macrophages and starts the initiation of skeletal muscle repair [1]. Therefore, we chose time points 5, 14 and 28 in order to evaluate the muscle regeneration as early as possible (day 5), and at intervals corresponding to progressive (day 14) [31] and complete (day 28) muscle regeneration (muscle mass recovery and myofiber cross-sectional area). Particularly, day 28 was selected because in our pilot study [5], we observed that muscle mass needed more than 24 days to recover completely.

### Quantification of muscle regeneration

Muscle regeneration was quantified by measuring the percentage of centrally nucleated fibers (CNF), the cross-sectional area, and the recovery index (% *sham* value) for both muscle mass and cross-sectional area at studied time points following injury. The interstitial area of regenerating muscles (IA) was also measured at days 14 and 28 after bupivacaine injection. Central nucleation is a good morphological index of muscle regeneration as it is a hallmark of fibers undergoing regeneration [32]. For myofiber cross-sectional area (MCSA) measurements, three transverse sections (10 µm thick) from the midbelly portion of each frozen part of TA were stained with hematoxylin-eosin. Two non crossing images of each section were digitally captured at 20X magnification and the number of CNF was counted. Images were then analysed using ImageJ Analysis Sotfware Program (ImageJ, National Institutes of Health, Maryland, USA) to measure each MCSA. Individual MCSAs (on average 75 fibers per section totalizing more than 200 fibers per muscle) were summed to determine the total myofiber cross-sectional area (TMCSA). The total cross-sectional area (TCSA) was then traced and the IA was estimated by subtracting the TMCSA to the TCSA [33]. The recovery index is the ratio of the average TA mass or MCSA in the injured leg to the corresponding values for *sham*.

### Immunohistochemical analyses and cell counting

The concentration of inflammatory cells was quantified using the method previously described [2]. Briefly, three sections (10 µm thick) from each frozen part of muscles were blocked (in blocking buffer) for 1 h and incubated for 2 h with primary antibody, consisting of either mouse anti-rat W3/13 (CD43) (1:50, Serotec, Raleigh, North Carolina, USA) to identify neutrophils, or mouse anti-rat ED1 (CD68) (1:100, Serotec, Raleigh, North Carolina, USA) to identify ED1^+^ macrophages, or mouse anti-rat ED2 (CD163) (1:100, Serotec, Raleigh, North Carolina, USA) to identify ED2^+^ macrophages. Under aseptic conditions like the one used in the protocol, CD43 is neutrophil specific in skeletal muscle because eosinophils, basophils, monocytes and B- and T-lymphocytes are not present. Sections were then washed with phosphate buffered saline and incubated for 1 h with biotinylated rat anti-mouse-IgG (1:200, Vector Laboratories, Burlington, Ontario, Canada) as secondary antibody. Finally, after 30 min of incubation with horseradish peroxidase avidin D (Vector Laboratories, Burlington, Ontario, Canada), sections were revealed using diaminobenzidine chromogen (Cedarlane, Burlington, Ontario, Canada). Labeled cells, expressed as the number of cells per mm^3^, were counted at 400X magnification in two non overlapping areas of each section. To accurately count the inflammatory cell profiles, a numbered grid divided into 100 squares was used. The area of this grid was 0.0625 mm^2^ at 400X magnification. The grid was initially set in the lower right of the section and was systematically moved up one grip until reaching the upper limit.

### Western blot assay

Protein content was quantified using the BCA Assay Kit (Thermo scientific, Mississauga, Ontario, Canada). Western blot was performed according to standard immunodetection protocols to quantify the expression of COX-2, and of two muscle-specific proteins, MyoD and myogenin, which characterize muscle regeneration. Two days after injury, we examined the expression level of COX-2 which is an inducible inflammatory enzyme early expressed after muscle injury. Its expression may persist for up to 3 days post-injury [34]. The expression levels of MyoD and myogenin were measured at day 2 and day 5 post-injury, respectively. MyoD is usually up-regulated by day 2 to day 3 following injury [7], indicating that satellite cells are committed to duplicate. Myogenin is expressed at high level when differentiation and myotube formation are initiated from day 3 to 5 post-injury [7]. The protein suspension (50 µg) was electroseparated by either a 9% sodium dodecyl sulfate-polyacrylamide (SDS) gel for COX-2 or a 10% SDS gel for MyoD and myogenin. Proteins were then electrotransferred to polyvinylidene difluoride (PVDF) membranes which were washed with buffer and blocked with BSA (3%) for 2 h. Membranes were immunoblotted overnight at 4°C either with COX-2 (H-62: sc-7951, 1:200), MyoD (C-20: sc-304, 1:500) or myogenin (M-225: sc-576, 1:200) polyclonal rabbit IgG as primary antibody (Santa Cruz Biotechnology, Santa Cruz, California, USA), all diluted in BSA. GAPDH (Santa Cruz Biotechnology, Santa Cruz, California, USA) was used as a protein loading control. After rinsing, membranes were incubated for 1 h with HRP-linked-mouse anti-rabbit IgG polyclonal secondary antibody (sc-2357) (Santa Cruz Biotechnology, Santa Cruz, California, USA) at a dilution of 1:10 000 in BSA (3%). Bands were detected with ECL-plus Western blotting reagent (PerkinElmer Life and Analytical Sciences, Wellesley, Massachusetts, USA). Luminescence was captured (Fusion FX7, Montreal Biotech Inc., Montreal, Canada) and the optimal density of each band was determined, corrected for the background, and analysed using the BIO-1D advanced software (Montreal Biotech Inc., Montreal, Canada).

### Statistical analysis

Results are reported as mean ± SEM. Normality was verified according to the Shapiro-Wilk test and observed for all data. ANOVA was then performed using the MIXED procedure of the Statistical Analysis System (SAS Institute, version 9.2, Cary, North Carolina, USA). When diet and time effects were observed, data were compared across time points (uninjured, 2, 5, 14 and 28 days post-injury) using Fisher’s protected LSD post hoc multiple comparison procedure. A sample size of 8 rats per dietary group per time point was determined based on the efficacy of dietary cod protein at influencing muscle weight and inflammatory response [5] at a probability level inferior to 0.05. However, we removed one outlier from statistical analysis of most measured parameters (centrally nucleated fibers, muscle mass, MCSA, interstitial area, density of neutrophils, density of ED1^+^ macrophage and ED2^+^ macrophages); therefore values are presented for n=7-8 rats/group/time-point. The measurement of COX-2, MyoD and myogenin was made randomly on 3 rats/group. Results were considered statistically significant at p ≤ 0.05.

## Results

### Food intake and body weight gain

In accordance with our previous study, the rate of growth was significantly higher in rats fed cod protein than in rats fed casein (Table 2). Although food intake and initial body weight were similar between groups, cod protein led to higher body weight gain compared to either casein (p=0.002) or casein+ (p=0.041). Consequently, the cod protein diet showed a greater gross weight gain efficiency than the casein (p=0.0003) and the casein+ diets (p<0.0001).

**Table 2:** Pre-injury body weight and food intake of rats fed purified diets for 21 days.

**Variables**	C	CP	C^+^
Initial body weight (g)	153±2	152±2	154±2
Pre-injury body weight (g)	320±4	332±4	324±5
Pre-injury body weight gain (g)	166±3^b^	180±3^a^	170±3^b^
Mean food intake (g/d)	23.0±0.3	23.6±0.4	23.6±0.4
Gross weight gain efficiency	0.34±0.00^b^	0.36±0.00^a^	0.34±0.00^b^

C, casein; CP, cod protein; C^+^, casein supplemented with L-arginine, glycine, L-taurine and L-lysine.

Values are mean ± SEM (*n* = 40 rats/diet).

^a,^ ^b^ Values on the same line with the same letter are not significantly different (p≤0.05).

Gross weight gain efficiency is the ratio of gram of body weight gained on gram of food ingested.

### Effects of cod protein on morphological characteristics of bupivacaine-injured skeletal muscle

**Figure 1:**
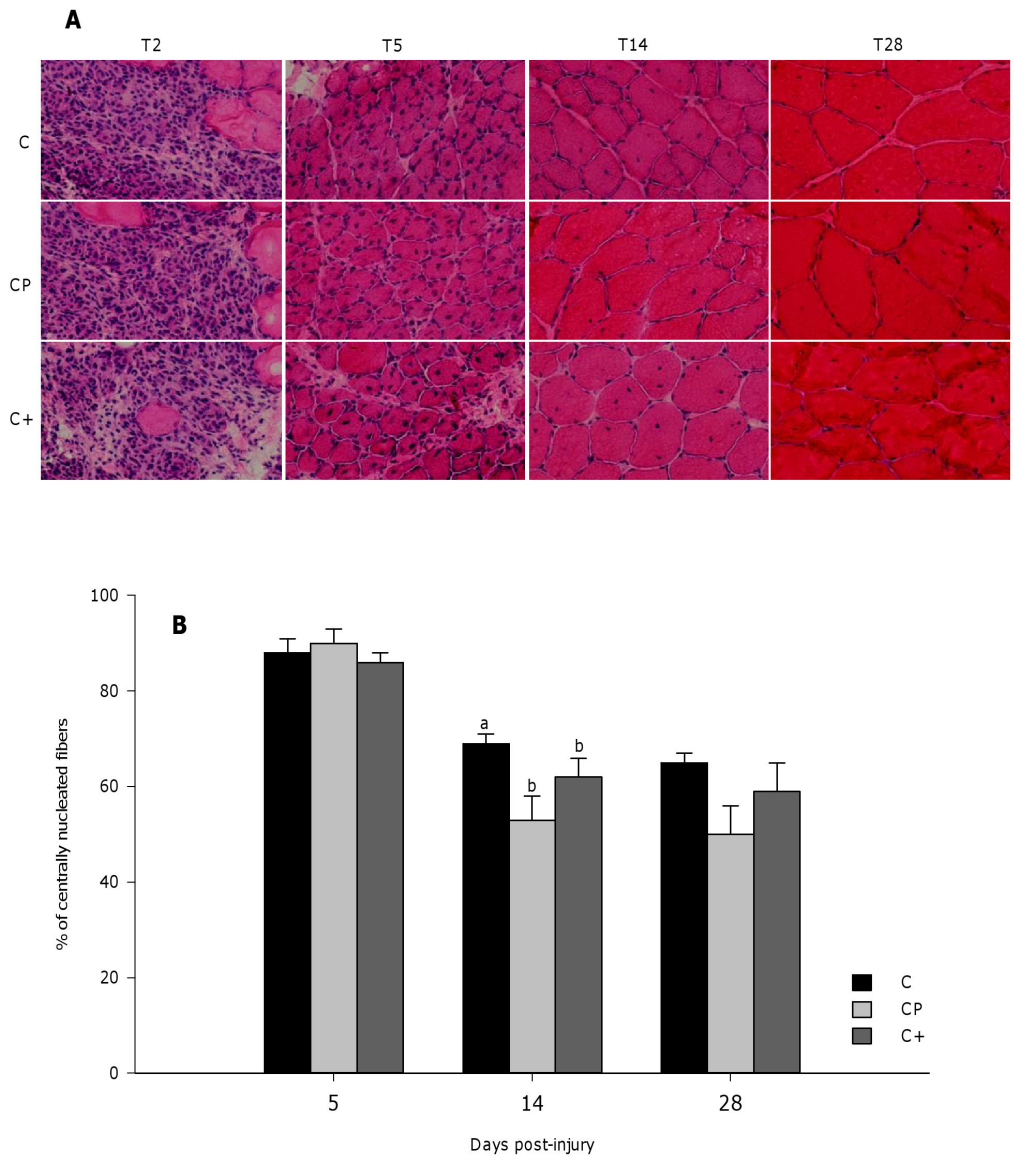
Representative H/E stained cross-sections and percentage of centrally nucleated myofibers. The number of centrally nucleated myofibers was counted in two non crossing H/E-stained sections of injured muscles collected at days 5, 14, and 28 post-injury. Representative H/E stained cross-sections and the percentages of centrally nucleated myofibers are shown in panels A and B, respectively. Data from panel B are expressed as a percentage of the total myofibers in the cross-sections. Values are mean ± SEM (n = 7-8 rats per dietary group/time point). Groups bearing different letters for a given time point are significantly different (p≤0.05). C, casein; CP, cod protein; C^+^, casein supplemented with L-arginine, glycine, L-taurine and L-lysine.

Two days after bupivacaine injection, TA muscle was edematous and a very significant proportion of the muscle fibers were necrotic and destroyed in all groups. By day 5 post-injury, necrotic debris were mostly cleared and replaced by regenerating fibers with a high proportion of centrally nucleated fibers (CNF) (Figure 1A). At day 14 post-injury, myonuclei significantly moved to the periphery of the muscle fiber (Figure 1A), leading to a decrease of central nucleation compared to day 5 post-injury (p<0.0001) (Figure 1B). At that time point, both the cod protein and casein+ diets reduced the percentage of CNF compared to the casein diet (p=0.025), while no significant difference was found between the cod protein and casein+ diets (Figure 1B). At day 28 post-injury, the proportion of CNF remained stable compared with day 14 and was not influenced by the diets.

**Figure 2:**
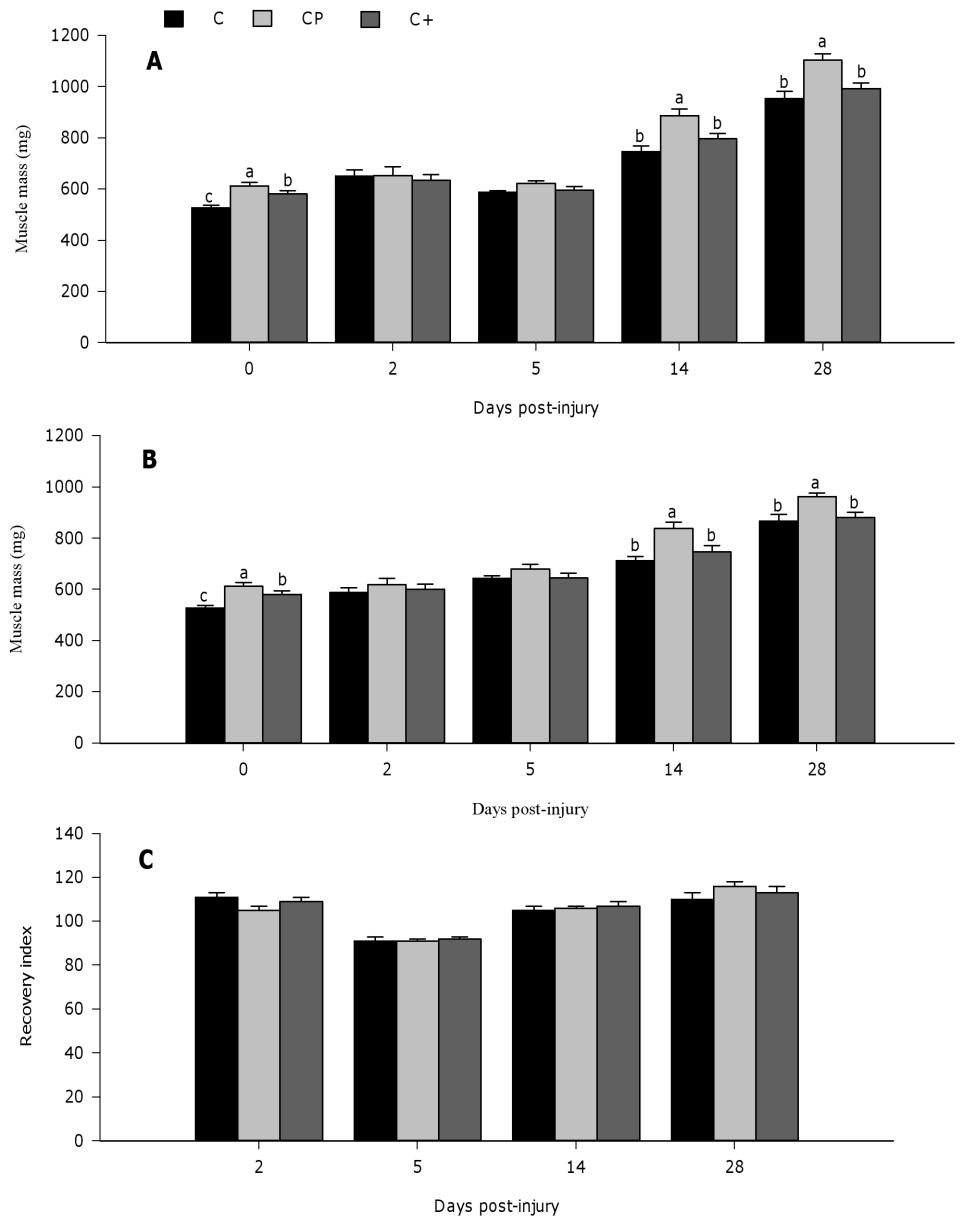
Muscle mass recovery of regenerating tibialis anterior muscle in rats. Both *sham* and bupivacaine-injured muscles were collected at days 0, 2, 5, 14, and 28 post-injury, and weighed. Muscle weights are presented for injured (A) and *sham* (B) muscles. A recovery index, as the percentage of injured relative to *sham* values, was calculated and presented for muscle mass (C). Values are mean ± SEM (n = 7-8 rats per dietary group/time point). Groups bearing different letters for a given time point are significantly different (p≤0.05). C, casein; CP, cod protein; C^+^, casein supplemented with L-arginine, glycine, L-taurine and L-lysine.

Figure 2 shows muscle mass in both injured (Figure 2A) and *sham* muscles (Figure 2B), and the recovery index (Figure 2C) at days 0, 2, 5, 14, and 28 post-injury. Prior muscle injury (day 0), the cod protein diet induced higher muscle mass compared with the casein (p<0.0001) and the casein+ diets (p=0.016). Muscle mass of the casein+ group was intermediary to, and significantly different from that of the casein (p=0.0002) (Figure 2A). At day 2 post-injury, muscle mass in the injured and *sham* muscle was not affected by dietary protein (Figure 2A, 2B, 2C). At day 5 post-injection, injured muscle mass tended to be higher in rats fed cod protein compared with those fed casein (p=0.066), whereas no effect of protein was observed in the *sham* muscle. The effect of cod protein on muscle growth and recovery was particularly visible at days 14 and 28 post-injury when the cod protein diet increased significantly the muscle mass value compared with the casein (day 14, p=0.002; day 28, p=0.001) and the casein+ (day 14, p=0.035; day 28, p=0.008) regimens (Figure 2A). Similarly, s*ham* muscle mass was also higher in rats fed cod protein compared with those fed casein and casein+ at day 14 (cod protein vs casein, p=0.005; cod protein vs casein+, p=0.039) and day 28 post-injury (cod protein vs casein, p=0.005; cod protein vs casein+, p=0.022) (Figure 2B).

**Figure 3:**
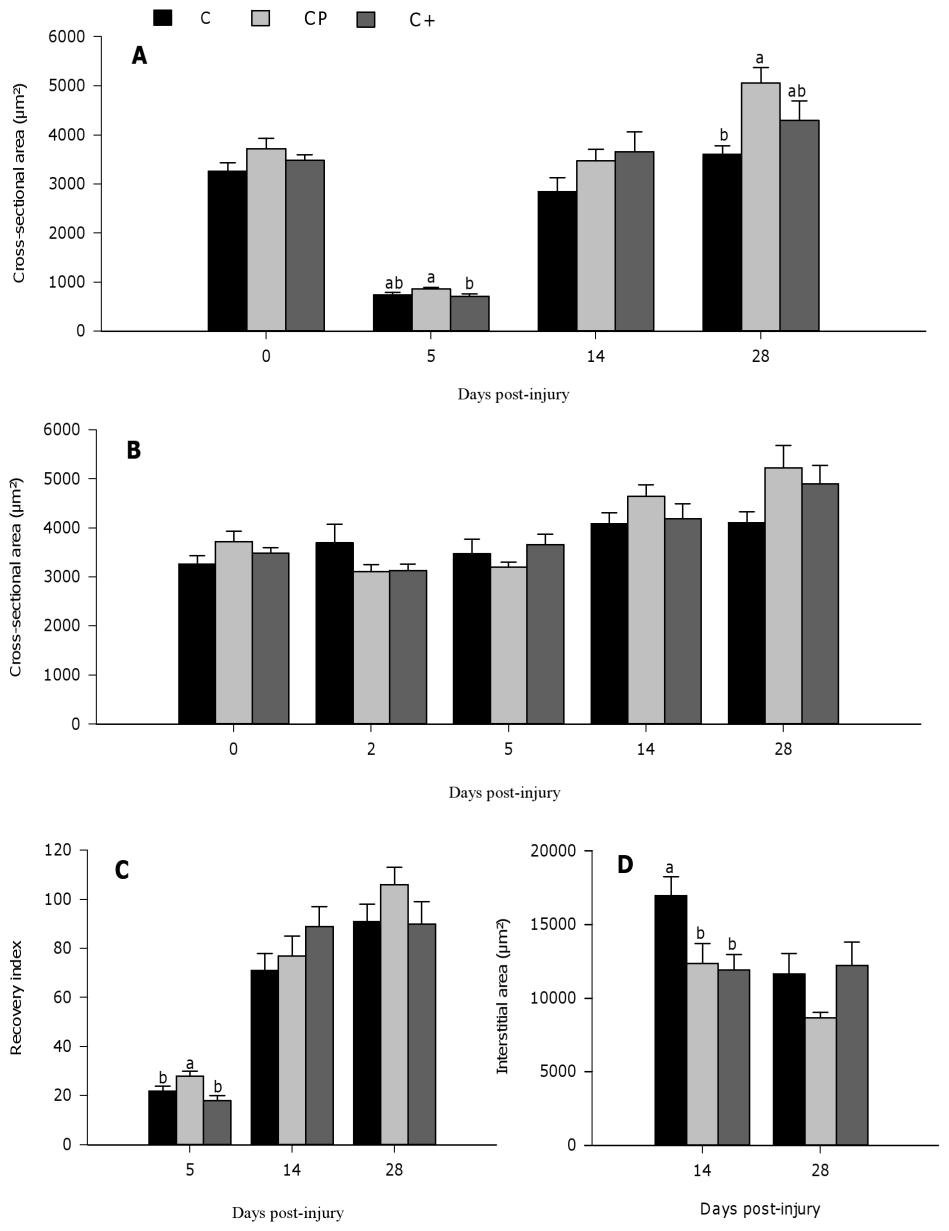
Muscle fiber cross-sectional area and interstitial area (µm^2^) of regenerating tibialis anterior muscle in rat. The myofiber cross-sectional area (MCSA) was quantified at days 0, 5, 14, and 28 post-injury in three H/E-stained cryosections from injured (A) and *sham* (B) muscles. The MCSA was not assessed at day 2 post-injury because of massive destruction of the myofibers in the necrotic area. Two images were captured at 20X from each cryosection and the cross-sectional area of each myofiber was traced using ImageJ Analysis Sotfware Program. A recovery index was calculated by dividing the mean MCSA for the injured muscle to the corresponding *sham* value (C). The total cross-sectional area (TCSA) was then traced in the injured muscle, and the interstitial area was quantified by subtracting the sum of individual MCSA to the TCSA at days 14 and 28 post-injury (D). Values are mean ± SEM (n = 7-8 rats per dietary group/time point). Groups bearing different letters for a given time point are significantly different (p≤0.05). C, casein; CP, cod protein; C^+^, casein supplemented with L-arginine, glycine, L-taurine and L-lysine.

The MCSA of regenerating fibers slightly tended to be higher in the cod protein-fed rats compared with those fed casein (p=0.093) at day 5 post-injury, while it was significantly increased compared with those fed casein+ (p=0.042) (Figure 3A). At day 14 post-injury, the MCSA of regenerating fibers increased significantly compared to day 5 post-injury (p<0.0001) and reached respective pre-injury levels in all groups. The positive effect of cod protein on MCSA was particularly evident at day 28 post-injury when compared with casein (p=0.012). MCSA values for rats fed casein+ were intermediary and not significantly different from that of rats fed either casein or cod protein. When compared to *sham* values (Figure 3B), cod protein feeding resulted in better MCSA recovery index than casein (p=0.028) and casein+ (p=0.001) feeding at day 5 post-injury (Figure 3C). At day 28 post-injury, although there was no significantly difference between dietary groups, MCSA of regenerating fibers of the cod protein-group reached the *sham* values, indicating a complete morphological regeneration, whereas those of the casein- and casein+- groups reached only 91 and 90%, respectively (Figure 3C). Consistent with these findings, the interstitial area of regenerating muscle was significantly reduced in the cod protein-group (p=0.039) and in the casein+-group (p=0.028) compared with the casein-fed counterparts at day 14 post-injury (Figure 3D).

### Cod protein modulates the inflammatory response through its high levels of arginine, glycine, taurine and lysine

**Figure 4:**
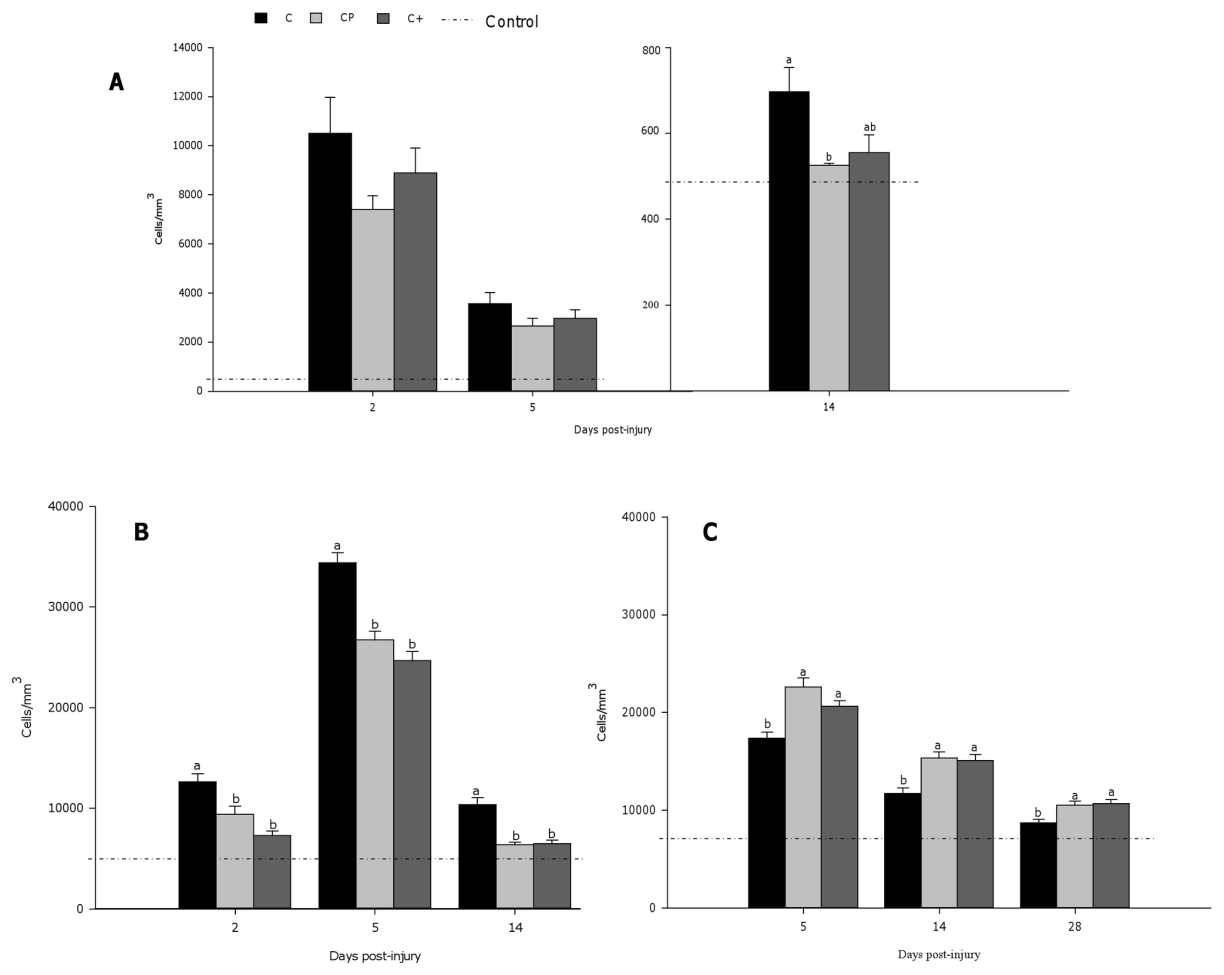
Time course of inflammatory cell accumulation in rat tibialis anterior muscle following bupivacaine injection. Transverse sections (10 µm) were immunoassayed with specific antibodies against neutrophils (W3/13) (A), ED1^+^-macrophages (B), or ED2^+^-macrophages (C). Labeled cells were counted at 400X magnification, and expressed as a number of cells/mm^3^. Values are mean ± SEM (n = 7-8 rats per dietary group/time point). As a control, values for neutrophils and macrophages at T0 are indicated by the dotted line. Groups bearing different letters for a given time point are significantly different (p≤0.05). C, casein; CP, cod protein; C^+^, casein supplemented with L-arginine, glycine, L-taurine and L-lysine.

**Figure 5:**
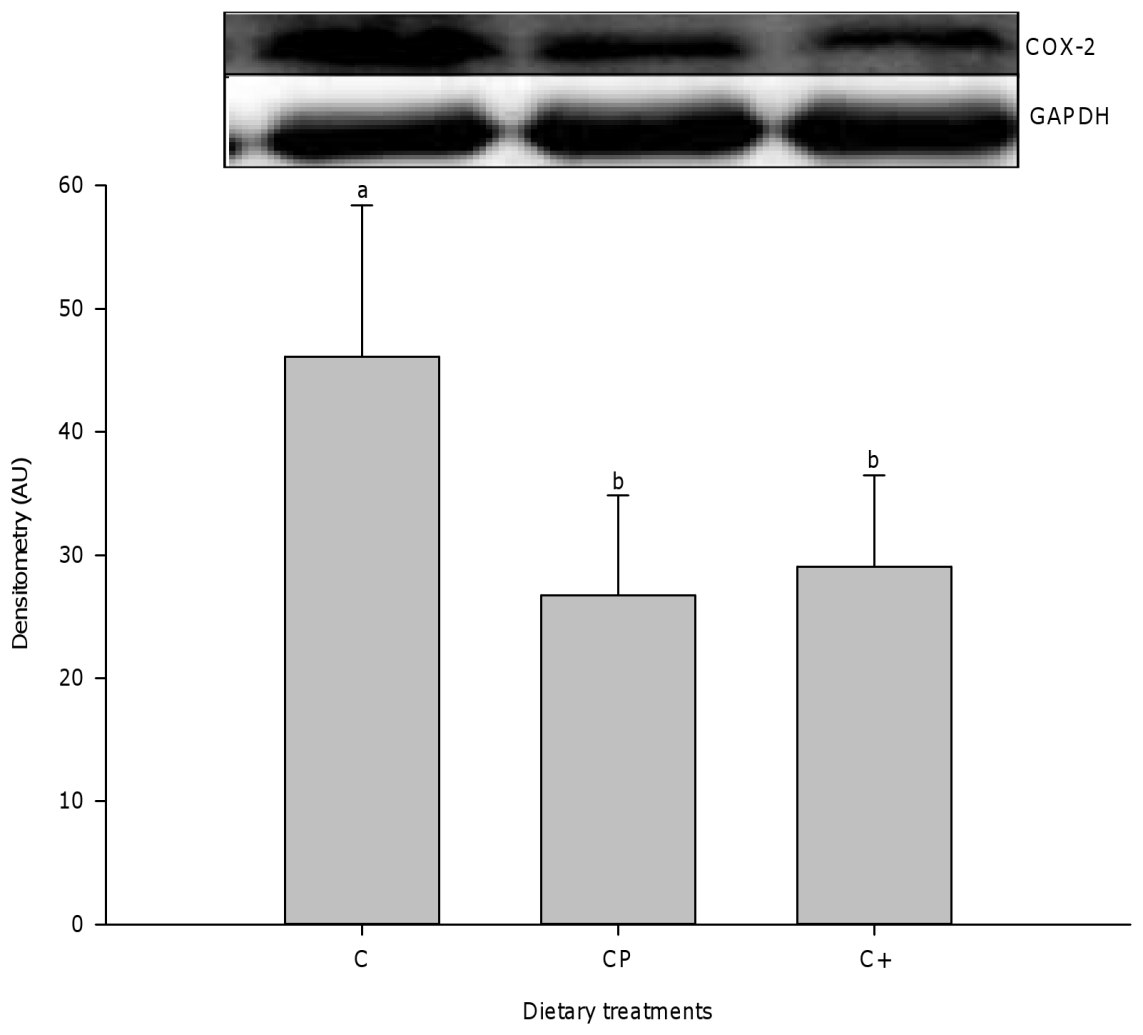
Expression level of COX-2 performed by immunoblotting. The protein level of COX-2, as a good marker of inflammation, was quantified in the tibialis anterior muscle of rats at 2 days post-bupivacaine injection. All values were corrected for GAPDH as a protein loading control (mean ± SEM, n = 3/group). Groups bearing different letters for a given time point are significantly different (p≤0.05). C, casein; CP, cod protein; C^+^, casein supplemented with L-arginine, glycine, L-taurine and L-lysine.

As a result of muscle injury, a robust inflammatory cell accumulation, dominated by neutrophils (Figure 4A), ED1^+^ macrophages (Figure 4B), and ED2^+^ macrophages (Figure 4C), was observed in all groups 2 and 5 days after muscle injury. However, cod protein and casein+ led to lower ED1^+^ macrophage accumulation compared to casein at day 2 (cod protein vs casein, p=0.012; casein+ vs casein, p<0.0001) and day 5 post-injury (cod protein vs casein, p=0.001; casein+ vs casein, p<0.0001) (Figure 4B). While neutrophil density decreased significantly across time points (p<0.0001) (Figure 4A), values for density of both ED1^+^ and ED2^+^ macrophages peaked at day 5 post-injury in all groups (Figure 4B, 4C). By day 14 post-injury, inflammation was greatly declined in all dietary groups, but the density of neutrophils and pro-inflammatory ED1^+^ macrophages was lower in cod protein- vs casein-fed rats (neutrophils, p=0.025; ED1^+^ macrophages, p<0.0001) and in casein+- vs casein-fed rats (neutrophils, p=0.060; ED1^+^ macrophages, p<0.0001) (Figure 4A, 4B). Consistent with these observations, cod protein- and casein+-fed rats exhibited higher density of ED2^+^ macrophages compared to their casein-fed counterparts at days 5 (cod protein vs casein, p<0.0001; casein+ vs casein, p=0.006), 14 (cod protein vs casein, p=0.001; casein+ vs casein, p<0.002) and 28 (cod protein vs casein, p<0.009; casein+ vs casein, p<0.005) post-injury (Figure 4C). In line with the smaller accumulation of pro-inflammatory macrophages, we also observed that both cod protein and casein+ markedly decreased the level of COX-2 compared with casein at day 2 post-injury (Figure 5) (cod protein vs casein, p=0.014; casein+ vs casein, p=0.029).

### Effect of dietary proteins on MyoD and myogenin content

**Figure 6:**
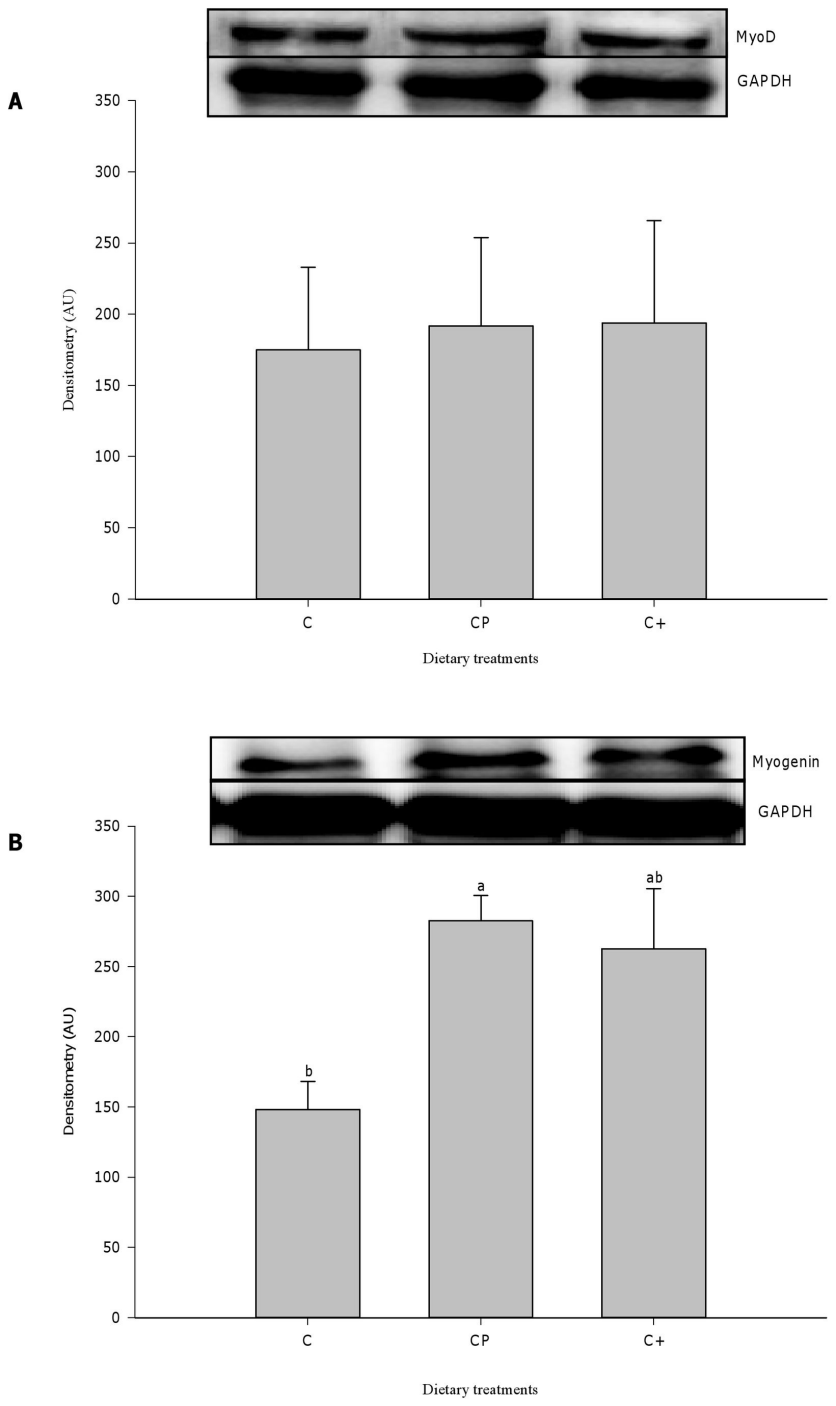
Expression level of MyoD and myogenin, performed by immunoblotting. The expression of MyoD (A) and myogenin (B), both are usually up-regulated during muscle regeneration, was measured in the injured muscles at day 2 and day 5, respectively. All values were corrected for GAPDH as a protein loading control. Results are expressed as a percentage of values obtained at day 0 (mean ± SEM, n = 3/group). Groups bearing different letters for a given time point are significantly different (p≤0.05). C, casein; CP, cod protein; C^+^, casein supplemented with L-arginine, glycine, L-taurine and L-lysine.

Quantification of protein level of MyoD and myogenin, sensitive markers of satellite cell activation-proliferation and differentiation, respectively [7], was also performed (Figure 6A, 6B). No significant difference was found for the myogenic regulatory factor MyoD at day 2 post-injury. On the other hand, the level of myogenin was up-regulated in cod protein-fed rats (p=0.037) and strongly tended to be increased in casein+-fed rats (p=0.062) compared with their casein-fed counterparts at day 5 post-injury.

## Discussion

We have previously shown that cod protein can decrease the accumulation of pro-inflammatory ED1^+^ cells at the site of injury after muscle trauma [5]. Data from the present study strongly suggest that the combination of arginine, glycine, taurine and lysine in cod protein was responsible for the modulation of inflammation in rat skeletal muscle after injury. Indeed, the addition of arginine, glycine, taurine and lysine to casein to achieve the levels found in cod protein, decreased ED1^+^ cell accumulation and COX-2 expression and increased the number of ED2 ^+^ cells as did cod protein when compared with casein. We further demonstrate an increase of myogenin expression and a faster recovery of muscle mass and MCSA in rats fed the cod protein diet compared to those fed the casein diet. While the arginine, glycine, taurine and lysine mixture tended to up-regulate myogenin expression, it had however no effect on muscle mass and MCSA; thus we conclude that this mixture of amino acids can specifically modulate inflammation but does not allow optimal recovery in terms of muscle mass.

Muscle injury was induced using bupivacaine as myotoxic substance. At day 2 after injury, muscle fibers were massively destroyed, as already shown by Politi et al [35]. At that point, muscle mass was similar in all groups, suggesting that the gain in mass due to edema has overcome the protein effect on muscle weight. However by day 5 post-injury, the regeneration process was clearly present and the influence of cod protein could be seen from that time point in the form of larger regenerating, centrally-nucleated fibers and an increased level of myogenin compared with casein. Globally, these data show that cod protein has a positive effect on both muscle growth and muscle recovery from injury. The effects of cod protein on recovery were mostly noted at days 14 and 28 post-injury when MCSA, the proportion of centrally-nucleated fibers and the interstitial space positively responded to the cod protein regimen. In particular, the positive correlation between muscle mass and MCSA at day 28 post-injury (r = 0.52, p = 0.013, n = 24) implies that the gain in muscle mass observed with the cod protein diet was partially due to the growth/recovery of myofibers rather than an expansion of intramuscular fat or connective tissue accumulation. We have previously demonstrated that rats fed the cod protein diet exhibited an increase in the body «protein:fat» ratio compared with their casein-fed counterparts [36]. In addition, we have noted that in high-fat fed rats, cod protein completely annihilated the deleterious effect of fat feeding on the insulin stimulated PI 3-kinase/Akt activity in skeletal muscle [37], a common metabolic disturbance also observed after an injury [26]. Evidences clearly indicate a central role of PI3-kinase/Akt generated signals, which act either in parallel with or downstream of muscle-specific transcription factors such as myogenin to govern the later stages of muscle differentiation [38,39]. Together, these observations suggest that cod protein, most likely via the stimulation of PI3-kinase/Akt activity, may also increase protein synthesis and decrease myofiber protein degradation [40], thereby improving muscle mass recovery. Further studies are needed to test whether the insulin and/or IGF-1/PI3-kinase/Akt pathways are involved for the benefits seen with cod protein.

Despite similar changes in muscle mass in both *sham* and injured muscles, the beneficial effect of cod protein was less evident in the *sham* leg with regards to the MCSA. These findings might result from local secretion of growth factors and upregulation of specific genes in the injured muscle, which normally occur during the regeneration process, thus improving the global dietary protein impact in the injured leg compared with the *sham* leg. In particular, injured skeletal muscles have been shown to release high levels of insulin-like growth factor-1 (IGF-1) compared with uninjured muscles [41]. This might subsequently result in enhanced IGF-1 signaling pathways stimulating muscle cell growth and proliferation to a greater extent in injured muscle compared to *sham*.

Beside its myogenic action, cod protein led to modulation of macrophage populations, namely a reduced ED1^+^ cell count, while increasing by more than 20% ED2^+^ cell accumulation (days 5, 14, 28) compared with casein. Immunologically, a general classification suggests that macrophages can be divided into two main subsets according to their specific functions: “classically activated” M1 macrophages, which are present in the inflammatory period and associated with phagocytosis and “alternatively activated” M2 macrophages, accumulating at the site of injury once necrotic tissue has been removed and participating in the regeneration and remodelling process. While not exactly based on the same identification markers, in rats the classification of macrophages as ED1 or ED2 subtypes closely resembles the M1 and M2c phenotypes, respectively. ED1^+^ cells are phagocytic and can enhance muscle damage and dysfunction through producing excess cytotoxic molecules such as proteases, ROS and numerous pro-inflammatory factors (TNF-α, IL-1β, IL-6, PGE-2…) [2]. However, the ED2^+^ phenotype is known to stimulate satellite cell proliferation and differentiation through the release of growth factors [42,43], and to promote resolution of inflammation. Interestingly, changes in immune cell types and counts in the cod protein group are supported by a 42% reduction in the level of COX-2, which is responsible for the production of pro-inflammatory prostaglandins, at day 2 post-injury compared with the casein group. These findings suggest that cod protein may down-regulate the production of COX-2-dependent inflammatory mediators [2,44]. In support of this, we and others have also reported convincing evidence regarding the anti-inflammatory actions of fish proteins such as reduction of TNF-α expression in cultured human macrophages [13] and of both TNF-α and interleukin-6 expression in adipose tissue of high-fat/high-sucrose-fed rats [12]. Such effects might result in a reduced necrotic zone and an accelerated switch of pro-inflammatory ED1^+^ cells toward the anti-inflammatory phenotype (ED2^+^) in the cod protein group [42,45-47].

One key observation of the present work is that the casein diet supplemented with a mixture of arginine, glycine, taurine and lysine to match their respective levels in the cod protein diet mostly mimicked the modulation of inflammation seen with the cod protein diet. Indeed, the casein+ diet reduced the quantity of ED1^+^ pro-inflammatory cells and COX-2 expression and increased the accumulation of ED2^+^ anti-inflammatory cells at the site of injury compared with the casein diet. This resulted in a strong tendency to up-regulate the expression of myogenin at day 5 post-injury. Thus, in accordance with our first hypothesis, these findings strongly suggest that high levels of arginine, glycine, taurine and lysine in cod protein might explain its beneficial effect on inflammation. Possible mechanisms explaining this modulation of inflammation could include a slower recruitment of monocytes from the microcirculation likely attributed to high levels of arginine and glycine in casein+ and cod protein. Indeed, evidence exists that arginine can inhibit chemotaxis, leukocyte rolling, and transmigrating out of the vessels through a nitric oxide-dependent mechanism [48-50]. Arginine supplementation has also been shown to reduce the accumulation of neutrophils by 66% and macrophages by 33% in endotoxin-induced lung injury [21] and renal allografts [23], respectively. On the other hand, glycine has been shown to decrease mRNA peak expression of ICAM and MCP-1 in rodent postoperative ileus, leading to reductions in both neutrophils and macrophages [22]. A second possible mechanism is that high levels of arginine, glycine and taurine in cod protein and casein+ can decrease the production of distressing inflammatory markers, attenuating the chemotactic recruitment of new leukocytes. Hnia et al [15] have indeed reported protective effects of L-arginine on muscle cell membrane integrity through a decreased TNF-α (-62%), IL-1β (-56%) and IL-6 (-54%) secretion. In addition, glycine has been shown to reduce the expression of COX-2 and the production of TNF-α, IL-6, IL-1β, and to protect muscle cells from apoptosis [17]. Furthermore, taurine may act as an anti-inflammatory agent through the formation of taurochloramine which inhibits the production of IL-6, IL-8, IL-2, TNF-α, and PGE-2 [51] and ROS generation [16,18,20], thereby decreasing muscle cell damages.

In contrast, with regards to our second hypothesis, casein+ did not improve muscle mass recovery in the same way as cod protein despite its similar effect on inflammation compared with cod protein. It is likely that the lower values for muscle mass and MCSA in casein- and casein+- fed rats were related to the lower content of essential amino acids, in particular sulfur amino acids [52] and threonine [36], in casein compared to cod protein. In the present study, casein had 16% less methionine and 11% less threonine than cod protein (Table 1). In a similar way, as a result of casein supplemented with the amino acid mixture, casein+ provided 23% and 20% less methionine and threonine than cod protein (Table 1), respectively. Therefore casein+ supplied lower amounts of methionine and threonine than cod protein, resulting likely in reduced anabolic and/or anti-catabolic potential [52-54] and slower muscle mass during the recovery period. Further research is needed to study the influence of a supplementation of casein with sulfur amino acids and threonine in addition to arginine, glycine, taurine and lysine on muscle mass regeneration.

A limitation of this study is that some parameters were presented only at what was seen to be the most relevant time points according to previous studies [1,8,30,31]. Therefore, we cannot exclude the possibility that effects at other time points would have been observed. For instance, the time point that we selected for MyoD measurement (day 2 post-injury) was not optimal to observe differences between dietary proteins. Day 3 post-injury would be more appropriate. Moreover, we were not able to detect serum inflammatory markers (TNF-α, IL-6) at the chosen time points. In future studies, we will extend sampling to other time points (e.g. ≤24 h post-injury) to detect changes in cytokines in response to dietary protein feeding. Measurement of cytokines will be also made directly in muscle lysate because the cytokines produced locally by the injured muscle might be diluted drastically upon reaching blood circulation.

In summary, we showed that the effect of cod protein on immune cell accumulation was mimicked by supplementation of casein with a mixture of arginine, glycine, taurine, and lysine, indicating that the high levels of these amino acids in cod protein may account for its anti-inflammatory effect post-injury. However, this amino acid mixture fails to induce a generalized effect on muscle mass recovery. It remains to delineate to what extent each of the supplemented amino acids produced anti-inflammatory effects and which mechanisms were behind these effects. Finally, future studies are needed to investigate the functional outcome of morphological changes observed with cod protein in order to design targeted nutritional strategies for the management of inflammation and muscle regeneration in athletes, and in patients and elderly people with inflammatory musculoskeletal diseases.
